# Validation of the Pediatric Canadian Triage and Acuity Scale at the Emergency Department of a Tertiary Children’s Hospital in Costa Rica

**DOI:** 10.7759/cureus.16191

**Published:** 2021-07-05

**Authors:** Xiomara Campos-Gómez, Natalia Martínez-Lara, Alicia Juncos-Moyano, Adriana Yock-Corrales

**Affiliations:** 1 Emergency, Hospital Nacional de Niños “Dr. Carlos Sáenz Herrera”, CCSS, San Jose, CRI; 2 Emergency, Hospital Maximiliano Peralta Jiménez, CCSS, Cartago, CRI

**Keywords:** triage, pediatrics, emergency department, children, ctas

## Abstract

Background: The Pediatric Canadian Triage and Acuity Scale (PedCTAS) is a recognized system that prioritizes care by the severity of illness. The goal of this study was to describe and analyze the results from the implementation of the PedCTAS in a tertiary children’s hospital in Costa Rica.

Methods: This was a retrospective observational study of children presenting to the emergency department (ED) from 1st January to 31st December of 2019 in the only children’s hospital in Costa Rica. Outcome measures were hospitalization, ICU admission, waiting times from triage to physician time (TPT), left without being seen (LWBS), length of stay (LOS), in relation to the triage level, and final disposition.

Results: A total of 93,001 patients were admitted to the ED. The proportion for hospitalizations according to triage category was 85.3%, 40%, 14%, 4.3%, and 2% for patients triaged at CTAS levels I, II, III, IV, and V respectively. A total of 2045 (2.19%) patients were LWBS. Some 585 (0.62%) patients were admitted to ICU. Median TPT for each category was for levels I:12 min, II:20 min, III:22 min, IV:34 min, and V:54 min. The LOS in the ED patients triaged as levels I and II stayed longer and the mortality rate was also higher in patients classified as levels I and II. The mortality rate was for level I patients 44.2% (23 patients) and level II 1.4% (8 patients).

Conclusions: This study shows evidence of validation of the PedCTAS in a developing country in Latin America. Implementation of a validated triage tool in our country helps us to provide improvements in the care of pediatric patients in the ED.

## Introduction

Triage systems in the emergency departments (EDs) are a complex process that provides an efficient system to prioritize the more severely ill patients; and the pediatric triage represents a challenge to the health personnel due to communication difficulties and great variability of parameters like vital signs, epidemiology, and clinical presentations of various disease [1].

The variety of triage systems used in the pediatric population around the world is wide, with systems including from three-level to five-level categories. The most used triage systems validated in the pediatric population in high- and middle-income countries are the Australian Triage Scale (ATS), the Canadian Triage and Acuity Scale (CTAS), the Manchester Triage System (MTS), and the Emergency Severity Index (ESI). They are five-level triage systems and studies have been performed in their respective home countries, in the adult population and there are no studies in low resource settings about the use and validation of these triage systems [2]. Another classification system, which is the Emergency Triage Assessment and Treatment (ETAT) of the World Health Organization (WHO) is a three-level triage system used in some low-resource settings as a triage system. The ETAT is a well-known triage system and adaptations have been made in some countries like South Africa [3-4].

The Pediatric CTAS (PedCTAS) is a triage method that has been used widely in Canada and other countries since 2001 [5], and previous studies have evaluated the validity of the CTAS for children. A few years ago, a prospective study was conducted among nine EDs of the Pediatric Emergency Research Canada network to evaluate the validity of the CTAS [6]. This study demonstrated a good correlation between triage level and different markers of severity, but it had two limitations, the small sample size (1464) and that they excluded in the analysis patients triaged at level 1 (resuscitation). Also, a study in Israel concluded that PedCTAS was good in predicting hospitalization admission to the ICU, proportions of left without being seen (LWBS), and length of stay (LOS) in ED [7]. 

Costa Rica has a public health system made up of 29 hospitals that include general and specialized hospitals. Since 2014, all the public hospitals with EDs (25) in Costa Rica incorporated the CTAS as a triage system in the EDs; and in the only tertiary pediatric hospital in our country, the PedCTAS was implemented in July 2018. This is the only referral and teaching pediatric center in the country for more than one million children under 13 years of age with an annual ED consultation of 98,000 and 10,000 admissions. Costa Rica is ranked 58th with a per capita gross domestic product (GDP) of US$ 12,238 for 2019, as a middle-income country [8]. The objective of this study was to determine the validity of the CTAS in the ED of the only tertiary pediatric hospital in our country; this is the first study in Latin America validating a triage system developed by a high-income country.

## Materials and methods

Study design 

This was a retrospective observational study of all children presenting to the ED from 1st of January to 31st of December of 2019 in the only referral and teaching pediatric center in the country for more than one million children under 13 years of age with 310 hospital beds and around 98,000 ED annual visits. The study was done by using the computerized database extracted from the electronic health record. The study was approved as an administrative study by the Deputy Director of the Hospital and did not require Institutional Board review. The primary outcome measures were hospitalization and ICU admission according to triage level and secondary outcomes included waiting times from triage to first doctor evaluation, the proportion of leaving the ED without being seen (LWBS), length of stay (LOS) in the ED in relation to the triage level, and final disposition. LOS was defined as the time from patient registration to the final disposition made by the ED physician. The rate of revisits at 72 h was determined for each triage category and the main presenting complaint was defined as the Canadian Emergency Department Information System (CEDIS). Triage to physician time (TPT) meant the waiting time from triage until first assessment by the ED physician. Ideal TPT should be less than 5, 15, 30, 60, and 120 min for CTAS categories I, II, III, IV, and V respectively. Lastly, we also reported the mortality rate as per the triage category.

Data analysis

Data were extracted from the electronic health records of our Institution (EDUS = Expediente digital en salud). All data were entered into an Excel 2008 spreadsheet for Microsoft 365 MSO (Microsoft, Richmond, WA) and analyzed with Stata/IC 16 (StataCorp, College Station, TX) software. The data included were basic demographic information of the patients, triage-related information, triage category, waiting times, and discharge disposition. Descriptive analysis was made for all the variables included. Continuous variables were presented as mean and standard deviation (SD) (normal distribution) or median and interquartile range (IQR) (non-normal distribution).

## Results

During the study period, a total of 93,001 patients were admitted to the ED and triaged with the CTAS system. The distribution of patients per triage category were level I 520 (0.55%), level II 5,722 (6.14%), level III 18,981 (20.37%), level IV 44,384 (47.63%), and 23,394 (25.1%) as level V patients. The mean age of study patients was 3.5 years (SD = 1.4 years; median 3.2 years with IQR 1.3-7.3). Of all patients 55% were males. Characteristics of the patients and presenting complaints according to CEDIS are seen in Table 1.

**Table 1:** Characteristics of patients admitted to the ED during the study period. Data are represented as n (%) unless otherwise indicated. Level 1 – patient requires immediate evaluation and care. Level 2 – patient requires evaluation and care within 15 min. Level 3 – patient requires evaluation and care within 30 min. Level 4 – patient requires evaluation and care within 60 min. Level 5 – patient requires evaluation and care within 120 min.

Variable	n	%
Age (mean, SD)	3.5	1.4
Sex		
Male	51161	55
Female	41832	44.9
Main Presenting Complaints (CEDIS)		
Fever	13942	15
Cough/Congestion	11360	12.2
Abdominal pain	6105	6.5
Upper extremity trauma	5476	5.8
Dyspnea	3597	3.8
Level of Triage (PedCTAS)		
Level I	520	0.55
Level II	5722	6.14
Level III	18981	20.37
Level IV	44384	47.63
Level V	23394	25.2

**Figure 1:**
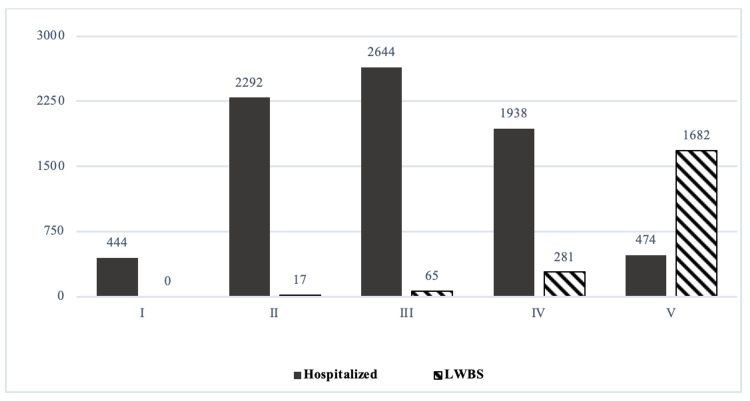
Number of patients hospitalized and LWBS according to triage level during 2019. LWBS, left without being seen

Regarding final disposition, a total of 83,507 patients were discharged (89.6%); 7,318 patients were hospitalized (7.85%), 1,815 patients were referred to another hospital center (1.94%), and 36 patients died (0.38%) in the ED. A total of 585 (0.62%) were admitted to ICU and 2045 (2.19%) were left without been seen. The proportions for hospitalizations according to triage category were 85.3%, 40%, 13.9%, 4.3%, and 2% for patients triaged at CTAS levels I, II, III, IV, and V, respectively. The proportion of children LWBS was higher among children triaged in lower acuity triage categories as seen in Figure 1. A total of 275 (52.8%) and 181 (3.1%) patients admitted to ICU were categorized as levels I and II, respectively; 71 (0.37%) were level III patients and 58 (0.08%) were categorized as levels IV and V patients.

The TPT varied according to the triage category. Fifty-eight percent of patients level I and 23.1% of patients level II were seeing in <10 min, 76.9% of patients level III were seeing in <30 min, 85.2% of patients level IV were seeing in <60 min, and 86.4% of patients level V was seeing in <120 min. Median TPT for each triage category was for level I - 12 min (IQR 3-12), level II - 20 min (IQR 6-28), level III - 22 min (14-34), level IV - 34 min (IQR 16-44), and level V - 54 min (IQR 16-75). As expected, TPT varied by triage category with level IV and V patients having the longest TPT times. More than half of all category patients met the set CTAS standards. The mortality rate was also higher in patients classified as level I and II as expected. The mortality rate was for level I patients 44.2% (23 patients), level II 1.4% (8 patients), level III 0.21% (4 patients), level IV 0.02% (1 patient), and no level V patients died during the study period. 

**Figure 2:**
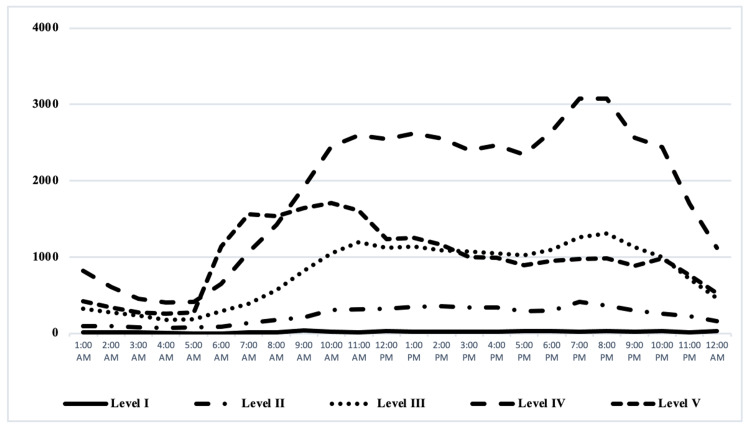
Time of admission of patients in the ED during 2019. Time of admission of patients according to CTAS triage category CTAS, Canadian Triage and Acuity Scale

In our study sample, the admission time varied according to the level of triage. In patients triaged as level I there was not a defined trend in time of admission. As shown in Figure 2 level IV patients were admitted to the ED more frequently in the evening hours (4 pm-11 pm) and category five patients consulted more frequently in the morning hours (6 am-12 pm); in general, the admission of patients during the early morning hours decreased in all categories.

**Figure 3:**
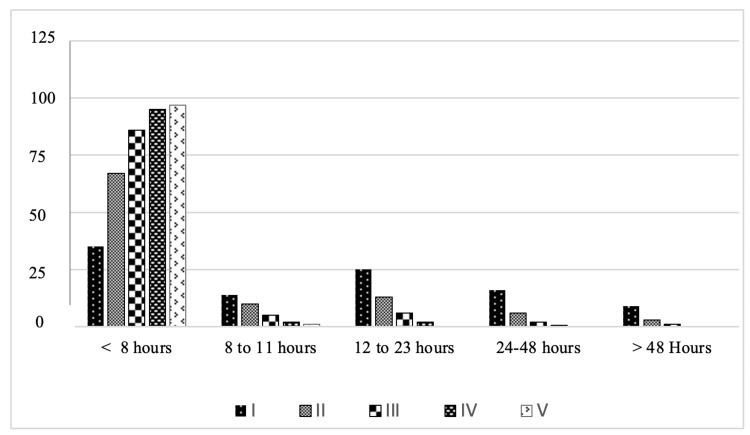
Length of stay in the ED according to the CTAS triage category. Length of stay in the ED according to the CTAS triage category percentage per level ED, emergency department; CTAS, Canadian Triage and Acuity Scale

The LOS in the ED varied as well in patients with different levels of triage; with patients triaged as levels I and II stayed longer in the ED compared with those with less acute PedCTAS levels (Figure 3). More than 95% of patients levels IV and V had a LOS of < 8 h and 50.7% of patients level I stay in ED for more than 12 h. 

The rates of revisits at 72 h from the first admission at ED were higher in levels I and II patients than in lower acuity levels. The rates of revisits at 72 h were 4.8%, 3.4%, 2.4%, 1.6%, and 2.4% for patients triaged at CTAS levels I, II, III, IV, and V respectively.

## Discussion

The validation of a triage system relies on the ability to discriminate different levels of urgency and it should correlate with resource use requirements and predict outcomes, including hospitalization and mortality rate [9-10]. For the validation of the triage system we compared the performance with the reference standard developed by experts and the association of levels of urgency with hospitalization, mortality rate, and length of stay in ED [11]; making this study one of the few studies of validation of the PedCTAS outside its country of origin and the first in Latin America [2, 7, 12].

We found that patients triaged at more acute PedCTAS levels had a higher chance for hospitalization and ICU admission as we expected. The first Canadian multi-center study evaluating the triage system of Gravel et al. found a strong association between these markers and the CTAS triage levels [6]. Similar results were made available in studies outside Canada like the one published in Israel [7, 12-13]. In Allon et al. the proportion of patients transferred to ICU were 24.2%, 3.05%, 0.24%, 0.05%, and 0.05% for PedCTAS levels I, II, III, IV, and V, respectively [7]. We had a higher proportion (52.8%) of patient level I admitted to ICU and this could be explained by the fact that a great proportion of those patients were transferred from another facility with advanced airway devices or major trauma as our hospital is the only specialized children’s hospital in our country; and other studies found similar trends [5, 12-13].

In our study, the hospitalization rate was 85%, 40%, 13%, 4%, and 2% for PedCTAS levels I, II, III, IV, and V, respectively with a general rate of 7.85%; in the study of Elkum et al., their hospitalization rate was of 6.7% very similar to our findings and different from Allon et al. that published a rate of 23.6% for hospitalized patients [7, 12]. The reason could be due to the differences in the management of patients in different contexts of the world and that can affect the decision to hospitalize a patient or not.

Some of our findings differ from previous studies; we found a higher proportion of level V patients (25.1%) compared to 16.1% in the study by Elkum et al. [12], 4.4% in Allon et al. [7], and 7% in Gravel et al. [5]. This finding could be due to the fact that we have patients who are scheduled a few days later for culture results assessment or review of laboratory tests performed in the ED; and according to CTAS, these patients are classified as level V. 

Patients with higher acuity levels had greater LOS; this could be secondary to a full ICU with no available beds to transfer patients from ED and also patients with levels I and II categories that might require more time for stabilization and management of their critical condition. Our data were according to expectations and are aligned with CTAS objectives according to what is established from the first studies about CTAS in pediatric patients in Canada [6].

Our study had some limitations to address and the main limitation of this study relates to the fact that the data came from only one institution and might be unable to generalize our findings to the whole EDs where pediatric patients are seen. However, this is the only pediatric referral hospital in our country and can reflect the reality of the performance of this triage tool in our pediatric setting. Other limitations result from the retrospective nature of the study, and we analyzed patients presenting only during a one-year period. 

## Conclusions

This study shows evidence of validation of the PedCTAS in a developing country in Latin America. We found that patients categorized as level I and II needed to stay longer in ED, hospitalization and ICU admission was higher; and the mortality rate was also higher as expected. Implementation of a validated triage tool in our country helped to improve the flow of patients inside the ED and reduced our waiting time of physician, especially for the high-level acuity patients.

Periodical reviews on the application of the PedCTAS in our hospital will be necessary to ensure quality data and detect cases of under or over triage. This study shows that care of pediatric patients in the ED can improve with the implementation of validated triage systems in other countries; this will give a better response to the recognition and early management of critically ill pediatric patients in our country and will stimulate other countries in our regions to implement a triage system like this one.
